# Suppression of ERECTA Signaling Impacts Agronomic Performance of Soybean (*Glycine max* (L) Merril) in the Greenhouse

**DOI:** 10.3389/fpls.2021.667825

**Published:** 2021-05-11

**Authors:** Yasmin Vasques Berchembrock, Flávia Barbosa Silva Botelho, Vibha Srivastava

**Affiliations:** ^1^Division of Agriculture, Department of Crop, Soil, and Environmental Sciences, University of Arkansas, Fayetteville, AR, United States; ^2^Department of Biology, Federal University of Lavras, Lavras, Brazil; ^3^Department of Agriculture, Federal University of Lavras, Lavras, Brazil

**Keywords:** ERECTA family, Δ*Kinase*, agronomic performance, drought tolerance, abiotic stress, soybean

## Abstract

The ERECTA (ER) family of genes, encoding leucine-rich repeat receptor-like kinase (RLK), influences complex morphological and physiological aspects of plants. Modulation of ER signaling leads to abiotic stress tolerance in diverse plant species. However, whether the gain in stress tolerance is accompanied with desirable agronomic performance is not clearly known. In this study, soybean plants potentially suppressed in ER signaling were evaluated for the phenotypic performance and drought response in the greenhouse. These plants expressed a dominant-negative *Arabidopsis thaliana ER* (*AtER*) called Δ*Kinase* to suppress ER signaling, which has previously been linked with the tolerance to water deficit, a major limiting factor for plant growth and development, directly compromising agricultural production. With the aim to select agronomically superior plants as stress-tolerant lines, transgenic soybean plants were subjected to phenotypic selection and subsequently to water stress analysis. This study found a strong inverse correlation of Δ*Kinase* expression with the agronomic performance of soybean plants, indicating detrimental effects of expressing Δ*Kinase* that presumably led to the suppression of ER signaling. Two lines were identified that showed favorable agronomic traits and expression of Δ*Kinase* gene, although at lower levels compared with the rest of the transgenic lines. The drought stress analysis on the progenies of these lines, however, showed that these plants were more susceptible to water-deficit stress as compared with the non-transgenic controls. The selected transgenic plants showed greater stomata density and conductance, which potentially led to higher biomass, and consequently more water demand and greater susceptibility to the periods of water withholding.

## Introduction

Water deficiency in different parts of the world could threaten sustainable planting of crops such as soybean (Kunert et al., 2016; Lesk et al., 2016). In dry conditions, plants keep their stomata closed, limiting carbon uptake for photosynthesis and, consequently, reducing grain yield. Exposure to water deficiency for a prolonged period results in changes in the metabolic pathways, reduction in organ size, oxidative damage, among other effects, which can compromise overall growth and even cause plant death (Nguyen et al., 2018; Leng and Hall, 2019). The perception of stress and transmission of signals, in order to activate adaptive response mechanisms, are important steps of stress tolerance in plants. One of the signal transduction pathways for transducing external signals is the mitogen-activated protein kinase (MAPK) cascade (Kaur and Gupta, 2005; Sinha et al., 2011) that are the key signaling modules downstream of receptor-like kinases (RLKs), like the ERECTA (ER) family of the leucine-rich repeat receptor-like Ser/Thr kinase (LRR-RLK) (Meng et al., 2012).

The ER family of receptor kinases plays important roles in cell–cell communication, regulating various aspects of plant development and physiological processes (Torii et al., 2003; van Zanten et al., 2009; Shpak, 2013). Modification in ER signaling in *Arabidopsis* leads to changes in stomata patterning, longitudinal growth, organ size, flower development, and plant architecture (Torii et al., 1996; Shpak, 2013), which in turn could affect plant’s ability to cope with the environmental stresses. Masle et al. (2005) found that *ER* is associated with the transpiration efficiency in *Arabidopsis* under well-watered and drought conditions, thus controlling photosynthetic and transpiration rates under different environments. Studying the leaf anatomical features, they suggested ER’s effects on stomatal density as one of the mechanisms of controlling leaf porosity and eventually transpiration efficiency. In another study, overexpression of *Arabidopsis ER* in rice was implicated in heat tolerance, independent of transpiration rate, suggesting a yet unknown physiological mechanism regulated by ER (Shen et al., 2015).

In order to study the function of ER in plant development, Shpak et al. (2003) expressed a truncated *Arabidopsis thaliana ER* (*AtER*) that encoded the receptor function but lacked the kinase domain (Δ*Kinase*), essentially acting as a dominant-negative receptor. The authors observed phenotypic changes similar to the loss-of-function *erecta* mutants and found a role for ER in cell size and proliferation during organ development. This Δ*Kinase* gene was later transformed into tomatoes with the aim to study the plant architecture and abiotic stress response (Villagarcia et al., 2012). The dominant-negative effect of Δ*Kinase* in tomato plants included reduced leaf size and number that presumably led to a greater tolerance to water deficiency. Interestingly, the fruit sizes and number were not reduced through the suppression of ER by Δ*Kinase* (Villagarcia et al., 2012). These observations suggested a biotechnology application of Δ*Kinase* in developing stress-tolerant crops. The sequence similarity and protein domain conservation between *AtER* and the four soybean ER homologs (GmERs) suggest functional conservation between the well-characterized *Arabidopsis* ER and the newly identified soybean ER genes (Du et al., 2017). Recently, Δ*Kinase* was transformed into soybean, and the resulting transgenic lines were analyzed for the phenotypic response (Shanmugam et al., 2020). As seen in the tomato plants, the expression of this Δ*Kinase* in soybean also resulted in smaller plants and reduced leaf size and number as well as drought and salinity tolerance as tested in the seedlings or the young vegetative plants (Shanmugam et al., 2020), confirming the functional role of *Arabidopsis* ER in soybean.

Generally, the most devastating drought episodes are the ones that occur during the reproductive phases of the plant. In soybeans, the period from the start of flowering stage through grain filling (R1–R5) can significantly affect the mean internode length, number of pods, number of seeds per pod, and seed weight (Fehr and Caviness, 1977; Desclaux et al., 2000). Farmers are generally able to avoid drought during the early vegetative stages by choosing a favorable planting date. However, potential drought later in the season during the flowering stages of the crop could be devastating. Therefore, development of drought-tolerant cultivars is important, and a combined conventional breeding and genetic engineering efforts may be needed to achieve this goal. Furthermore, since the expression of Δ*Kinase* influences phenotypic traits such as biomass and leaf size and number, the selection of plants through evaluations of agronomic traits is equally important for the practical application. This study investigated the effect of Δ*Kinase* on different phenotypic parameters of soybean plants in the greenhouse and tested the water stress response of the phenotypically superior plants expressing Δ*Kinase*. This analysis revealed a negative correlation of ER suppression and plant productivity, highlighting the role of ER in organ size and growth during vegetative and reproductive phases.

## Materials and Methods

### Plant Lines

Soybean transformations were carried out by the Iowa State University Plant Transformation Facility using the pTF101.1 vector (Addgene # 134770) harboring 8.0 kb *Arabidopsis ER* gene with a stop codon after the transmembrane domain and therefore lacking the kinase domain (Δ*Kinase*) (Shpak et al., 2003). The development of the transformation vector and the transgenic lines in Williams 82 background has been described earlier by Shanmugam et al. (2020).

### Plant Water Withholding Response

Twenty-one T_2_ plants, from three primary transgenic lines and one non-transgenic (empty-vector) control line, referred to as wild type (WT), were sown individually in 2.5-L pots containing a mixture of sphagnum peat moss and perlite (9:1), PRO-MIX LP15^®^, and Osmocote^®^ fertilizer (15N-9P-12K), which were previously weighed in order to standardize the amount of soil in each pot. The plants were kept in the greenhouse under normal light conditions with supplemental light between 8:00 am and 5:00 pm, when sunlight drops below 1,260 μmol/m^2^/s and temperature (22–27°C). All plants received normal water supply uniformly for 3 weeks. In the fourth week, all the pots were equalized in terms of volumetric water content, and for the analysis of water withholding response at the vegetative stage, the water was cut off and the pots were weighed daily until the irreversible senescence of the plant.

### Plant Phenotyping

Ninety-four seeds from 12 T_2_ lines comprising 10 transgenic, one WT, and one empty vector control (Supplementary Table 1) were sown individually in pots and maintained in the greenhouse under the same conditions as described above. All plants received normal water supply uniformly. At R5 stage (Fehr and Caviness, 1977), samples of the last trifoliate leaf developed in the main stem were harvested for RNA extraction. These samples were later used for correlating gene expression levels with the phenotypic performance. At physiological maturity, the agronomic performance of the plants was determined by evaluating the plant cycle from sowing to harvest (PC, days), shoot dry weight (SDW, g), shoot fresh weight (SFW, g), percentage dry matter of shoot (SDM, %), roots fresh weight (RFW, g), roots dry weight (RDW), number of grains per pod (GP), grain yield per pot (Y, g), and weight of 100 grains (100G, g). From that, 20% of the plants were selected, 10% of which had the best agronomic performance and 10% the worst, in relation to the favorable traits to the breeding program. These plants were ranked based on the sum of ranks (SR) selection index proposed by Mulamba and Mock (1978) according to the equation:

$$S\,R_{g}=\sum_{i\,1}^{n}i_{j}$$

Where:

*SR*_*g*_: SR index for each *g*th genotype;

*i*_*j*_: the genotype classification in relation to the *j*th trait.

### Water Stress Analysis

For the analysis of drought response at the reproductive stage, progenies from two T_3_ selected plants (4-3 and 4-23) based on SR together with WT were sown individually in pots and kept in the greenhouse under the same conditions as described above. In order to assess water-deficit tolerance, a completely randomized design in a factorial scheme (3 × 2) with three replications was used, each plot consisting of a pot with one plant. The evaluated treatments were the three genotypes in two water regimes, well-watered control, and water stressed (drought).

All pots received 180 mL of water daily until the R2 stage, characterized by the full bloom stage. From then on, due to the increase in water demand, the amount of water was increased to 360 mL daily in the control plants kept in the normal condition. However, the water-stressed plants had their irrigation suspended until they presented symptoms of wilting and visible loss of turgor. During the water stress experiment, the moisture in the soil of each pot was monitored using the HoldAll Soil Meter^®^. After 2 days of submitting the plants to water stress, the stomatal conductance was evaluated using a LI-6400XT portable photosynthesis system (LICOR Inc., Lincoln, NE, United States). Furthermore, the youngest trifoliate leaf of three plants from each genotype was collected for evaluation of stomatal density. The leaves were kept in 70% ethanol to remove the chlorophyll, and subsequently viewed in Keyence VK−X260 3D Laser Scanning Microscope with × 20 lens. Additionally, 1 cm^2^ of leaf was submerged in aqueous 0.1% Toluidine Blue-O (TBO) for 1 h and photographed under TrueChrome Metrics camera in × 40 lens. Finally, the phenotypic evaluation of the plants was carried out by estimating the following: plant height at 30 days after sowing (H30, cm), number of days from sowing to flowering (FLOR), the duration of the plant cycle as indicated by the number of days to maturity (PC), biomass shoot (SW, g), and root biomass (RW, g) obtained by weight, and SDM (%) and percentage dry matter of roots (RDM, %), number of nodes in the harvest (NH), GP, Y (g), and the 100G (g).

### Gene Expression Analysis

Total RNA was extracted from the first trifoliate leaf in seedlings of V2 stage (two nodes on the main stem with fully developed leaves), the youngest trifoliate leaf near the apex in R2 plants (full bloom stage with open flower at one of the two uppermost nodes on the main stem with a fully developed flower), and the young (1 cm) pods from the R4 plants (full pod stage with three-quarter-in.-long pod at one of the four uppermost nodes on the main stem) using TRIzol (Invitrogen—Life Technologies) reagent according to the manufacturer’s instructions and quantified by Nano-drop 2000 (Thermo-Fisher Inc.). The staging of the plants (V2, R2, and R4) is based on Fehr and Caviness (1977). All RNA samples were treated with RQ1-RNAse free DNase (Promega Inc.) for 30 min at 37°C, followed by the inactivation of DNase I by EDTA. All the DNase-treated RNA was quantified again, and a 1-μg DNAse-treated RNA was used for cDNA synthesis using PrimeScript RT reagent kit (Takara Bio., Mountain View, CA, United States). The real-time quantitative PCR was performed on the cDNA using TB green Premix Ex Taq II kit (Takara Bio., Mountain View, CA, United States) on Bio-Rad CFX 96 C1000 using Δ*Kinase ER* primers, 5′-GGACTTGTCCTACAATCAGCTAACT-3′ and 5′-TTGAACCAGTCAGCTTGTTACTGTGC-3′ or qGmACT11 5′-CCCTGGTATTGCTGACAGAATG-3′ and 5′-CACCGATCCAGACACTGTATT-3′. The amplification program consisted of 95°C for 30 s followed by 40 cycles of 95°C for 5 s and 60°C for 30 s. All samples were analyzed with two technical replications. The product specificity was verified by the melt curve analysis, and the relative expression of Δ*Kinase* was determined against the WT using the delta–delta C_*T*_ method (Livak and Schmittgen, 2001).

### Statistical Analysis

The phenotypic data were statistically analyzed by analysis of variance (ANOVA), and the Tukey test for comparisons among treatment means was applied, with 5% as significance level using R (R Core Team, 2017) and RStudio (RStudio Team, 2016) software. The Pearson’s correlation coefficient between the Δ*Kinase* expression and the phenotype traits was applied, and the Student’s *t*-test was performed adopting 5% significance level.

## Results

### Characterization of Transgenic Lines

Three different transgenic lines (#1, #2, and #3) expressing Δ*Kinase* gene were included in the study. T_2_ plants of these lines showed variable phenotypes based on plant height, leaf size, and number. Plants of line #1 were tallest with largest leaf size and number, while lines #3 and #2 plants were shorter with decreasing leaf size and numbers (Supplementary Figure 1). In a preliminary analysis of water-deficit response, transgenic plants together with the tissue culture-derived empty vector control plants, representing the WT controls, had irrigation suspended 3 weeks after sowing. Although all the pots started with the same water content (%), the pots of line #1 as well as WT controls, in the first 24 h of stress, reduced more than 40% of their initial weight. The plants from lines #1 performed similar to WT plants and died after 10 days of water cut off. Plants of line #3 generally lagged behind in the early phase of water stress but, similar to line #1, could not survive 10 days of water cut off. Line #2 plants, on the other hand, showed a gradual reduction in pot weight and survived water cut off without showing significant stress symptoms. This analysis suggested a correlation between plant vigor (leaf size and number) and water demand as bigger plants with greater foliage consumed water and irreversibly wilted during water withholding phase. Moreover, the morphological variabilities among transgenic lines highlight the importance of plant selection through phenotypic characterization for identifying genotypes of superior agronomic traits and potentially greater tolerance to water-deficit stress.

### Correlation of Δ*Kinase* With Phenotypic Characteristics of Transgenic Plants

**FIGURE 1:**
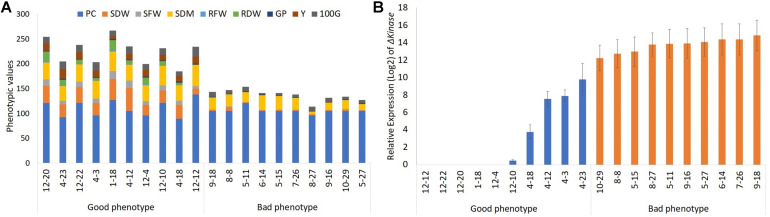
The effect of Δ*Kinase* on the phenotypic performance of soybean plants in the greenhouse. **(A)** Phenotypic values and **(B)** relative gene expression (log_2_ converted) in the top 10 (good phenotype) and bottom 10 (bad phenotype) plants ranked based on the agronomic characteristics: plant cycle (PC, days), shoot dry weight (SDW, g), shoot dry matter (SDM, %), roots fresh weight (RFW, g), roots dry weight (RDW, g), number of grains per pod (GP), grain yield (Y, g), and weight of 100 grains (100G, g).

A total of 94 T_3_ progeny plants, representing 26 line #1, 38 line #2, 23 line #3, 11 empty-vector controls, and three WT were grown in the greenhouse and evaluated for desirable agronomic traits (Supplementary Table 1). The evaluated traits were then used for estimating the selection index. The use of selection index in plant breeding allows combined evaluation of all available information, increasing the chances of selecting promising genotypes that contain a reasonable number of favorable alleles for different agronomic traits. The phenotypic evaluation of the best and lowest performers based on SR index (Supplementary Table 2) is shown in Figure 1A. The 10 genotypes that were associated with the best classification and, therefore, showed the lowest SR, were those with the desirable phenotypic characteristics for the crop (good phenotype). The 10 plants with the highest SR, on the other hand, are the genotypes with lowest agronomic performance within the group (bad phenotype). As expected, among the plants clustered in the “good phenotype” group are the non-transgenic WT (1-18) and the empty-vector controls (12-4, 12-10, 12-12, 12-20, and 12-22).

Next, the influence of Δ*Kinase* on the plant phenotype was determined by correlating its expression with the traits evaluated within the group of 20 soybean plants, representing “good” or “bad” phenotypes. This analysis revealed a detrimental effect of Δ*Kinase* expression on the agronomic performance of the plants included in this study. The Pearson’s correlation values were significant (*p* < 0.01) for all characters except PC (Table 1). The level of Δ*Kinase* expression, indicated by the mRNA abundance, showed a linear inverse relationship with the phenotypic traits of agricultural interest for the soybean crop, with Y as the most impacted character (Table 1).

**TABLE 1:** Pearson’s correlation value between the Δ*Kinase* expression and all traits evaluated in 20 soybean plants.

**PC**	**SDW**	**SFW**	**SDM**	**RDW**	**RFW**	**GP**	**Y**	**100G**
−0.171	−0.762*	−0.760*	−0.654*	−0.626*	−0.601*	−0.658*	−0.781*	−0.621*

*Significant by Student’s *t*-test at 1%. PC, plant cycle; SDW, shoot dry weight; SFW, shoot fresh weight; DMS, shoot dry matter; DWR, roots dry weight; FWR, roots fresh weight; GP, number of grains per pod; Y, grain yield per pot; 100G, weight of 100 grains.

Although the expression of the Δ*Kinase* appeared, in principle, to disrupt desirable phenotypic traits in soybeans, presumably due to the interference in the native ER signaling, it was possible to find three plants 4-3, 4-12, and 4-23 that showed good phenotypic performance, despite Δ*Kinase* expression (Figure 1B). The relative expressions of Δ*Kinase* in these plants were, respectively, 7. 9-, 7. 5-, and 9.8-fold above the background but lower as compared with that in the “bad phenotype” group of plants (14-fold).

To determine if Δ*Kinase* confers stress tolerance through a physiological process, independent of plant architecture modification, two representative genotypes based on their gene expression values (4-3 and 4-23), both originating from line #1, were selected for water stress analysis.

### Water Stress Analysis

**FIGURE 2:**
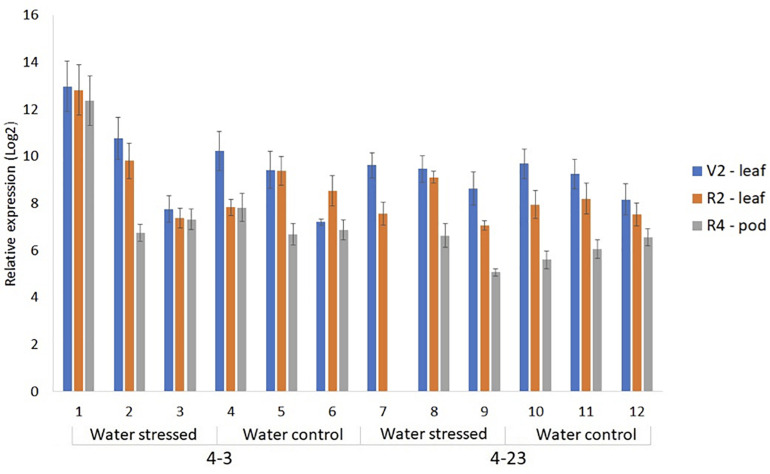
Relative expression of Δ*Kinase* in the progeny plants of 4-3 and 4-23 lines by RT-qPCR in the leaves at V2 stage and R2 flowering stage, and in 1 cm pods at R4 stage, in water-stressed and well-watered controls.

In soybeans, short-term and moderate water deficit during the vegetative stage may not reflect drastic reductions in grain yield since this period can be compensated by rainfall during the reproductive stage. However, in the reproductive period and, mainly during pod filling stage, drought can result in severe productivity losses (Oya et al., 2004; Xu et al., 2018). Therefore, six progeny plants of the lines 4-3 and 4-23, together with the WT control, were subjected to water stress at the R2 stage, when open flowers are observed at the uppermost nodes of the main stem (Fehr and Caviness, 1977). All plants were verified to express Δ*Kinase* in the trifoliate leaves at V2 stage, when fully developed trifoliate leaf is developed at the second node (Fehr and Caviness, 1977). This analysis showed a highly variable expression in the independent plants (Figure 2). Water stress was initiated by withholding water on the pots selected for the stress treatment, while continuing to water the control pots. Unexpectedly, all six transgenic plants showed drought symptoms characterized by visual loss of turgor in the first 48 h of water withholding. The transgenic plants showed greatest sensitivity to water deficit, with wilted and senescent leaves beginning to appear after just 1 day. In contrast, the WT plants did not show drought symptoms until the fifth day of water withholding. Based on this, the stress treatment on transgenic plants was altered by irrigating every other day, so that it would be possible to evaluate plant’s response to the stress. The well-watered control plants, on the other hand, continued to be watered every day. Although plants #1 and #2 had a rapid wilting response, they recovered to the point of producing pods and completing the cycle. However, plant #7 did not survive the stress treatment, and close to stage R4, died from water shortage. R4 stage is characterized by the full pod stage, showing 2 cm pods at one of the four uppermost nodes on the main stem with a fully developed leaf (Fehr and Caviness, 1977).

The expression of *ER* gene in *A. thaliana* is limited to the shoot, with a higher level of expression found in the reproductive parts (Torii et al., 1996; Shpak et al., 2004). Soybean genome contains four members of *ER* genes that show higher expression in the young leaves at vegetative and reproductive stages as well as in young pods of soybean plants (Supplementary Figure 2). Therefore, Δ*Kinase* expression was determined in the trifoliate leaves from V2 and R2 stages and 1 cm pods from R4. As seen in V2 stage, the Δ*Kinase* expression was variable from plant to plant; however, the expression was much lower in the pods from R4 stages, when compared with the V2 stage leaf (Figure 2). It should be noted that only R2 and R4 samples represented plants under stress and control conditions.

**FIGURE 3:**
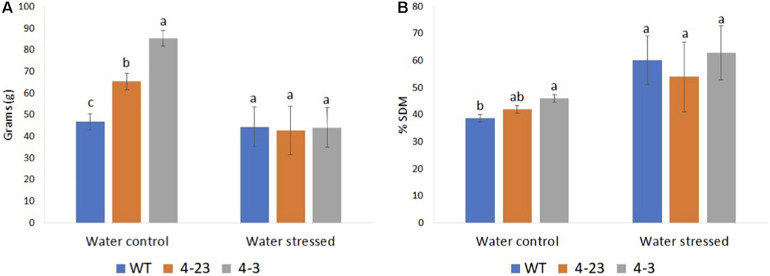
Shoot biomass of transgenic (4-23 and 4-3) and wild-type (WT) plants under water-stressed and well-watered control conditions; **(A)** shoot weight and **(B)** percentage dry matter of shoot. Different letters indicate significant differences (*p* < 0.05) between genotypes by the Tukey test (*n* = 3) under different water conditions. The error bars indicate ± SE.

Next, the agronomic traits were evaluated among the plants in this experiment. The traits affected by the different genotypes (*p* ≤ 0.05) were H30, SW and RW, SDM and RDM, and Y. For all other traits, including FLOR, PC, GP, NH, and 100G, no significant differences (*p* > 0.05) were observed between genotypes. Plant height was recorded on H30 during vegetative phase before stress treatment, while other traits were recorded at the end of cycle in both stressed and control plants. H30 of transgenic plants (4-3 and 4-23) was superior to the WT plants (Supplementary Figure 3). Next, the SW as well as the SDM was different between transgenic lines and WT under normal water conditions, especially for the #4-3 plants, which presented the highest averages for all these traits (Figures 3A,B). However, the SW in each genotype was significantly impacted by water stress, but a greater reduction was observed among transgenic plants (Figure 3A). Furthermore, although no significant difference was observed between the genotypes for grain yield, they showed a non-coincident response to different water conditions (Supplementary Figure 4). Once again, transgenic plants were impacted more severely by the stress as compared with the WT.

**FIGURE 4:**
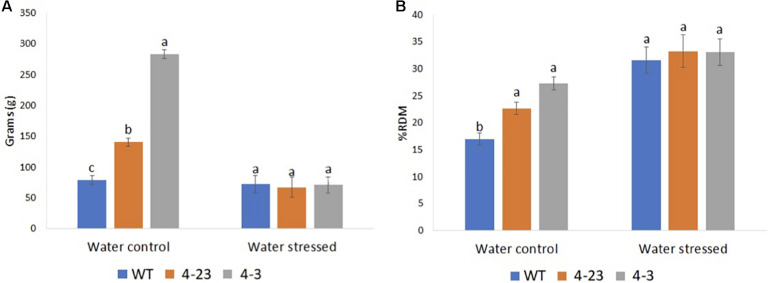
Roots biomass of transgenic (4-23 and 4-3) and wild-type (WT) plants under water-stressed and well-watered control conditions; **(A)** root weight and **(B)** percentage dry matter of root. Different letters indicate significant differences (*p* < 0.05) between genotypes by the Tukey test (*n* = 3) under different water conditions. The error bars indicate ± SE.

The interaction between the soil and the root system is also an important mechanism for plant’s response to drought since the roots grow to seek more humid regions of the soil. Interestingly, all the transgenic plants showed greater root biomass in the well-watered condition (Figures 4A,B and Supplementary Figure 5). However, although the RW and RDM were statistically different (*p* < 0.05), and higher in transgenic plants under the well-watered condition, these traits were not significantly different between transgenic and WT plants under stress conditions. For RW among WT plants, no major change was recorded between water conditions, and under stress, the plants maintained 91% of RW when compared with the irrigated plants. Transgenic plants (4-3 and 4-23), on the other hand, maintained just 25 and 48% in RW, respectively, when subjected to water stress (Figure 4A).

**FIGURE 5:**
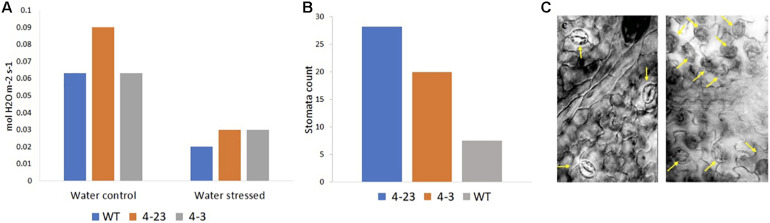
**(A)** Stomatal conductance in transgenic (4-23 and 4-3) and wild-type (WT) plants under water control and water stress conditions and **(B)** stomata density in transgenic (4-23 and 4-3) and WT plants under ×20 lens. **(C)** Stomata pattern in the abaxial leaf surface in WT (left) and 4-3 (right) transgenic plant under ×40 lens. The arrows in **(C)** point to the stomata.

The influence of the ER on leaf organogenesis, stomata patterning, and transpiration efficiency highlights its role in the coordination of transpiration and photosynthesis processes. With the expression of the Δ*Kinase* and the suppression of ER signaling in soybeans, an increase in stomatal conductance was expected that could result in greater water loss through transpiration. The stomatal conductance was higher in transgenic plants than WT (Figure 5A), and the microscopic analysis of the abaxial leaf surfaces revealed a higher stomatal density in transgenic plants (Figure 5B) with altered patterning consisting of clustered stomata in the transgenic plants as compared to the well-spaced stomata in the WT leaves (Figure 5C). A similar stomata pattern is found in the *Arabidopsis erecta* mutants that correlate with the increase in stomatal conductance (Masle et al., 2005; Shpak et al., 2005). Stomatal closure is an attempt by the plant to maintain optimum water content in the tissue, thus, under stress, a decrease in stomata conductance was observed for all plants (Figure 5A). However, the stomata closure apparently was not sufficient to prevent wilting in the plants of 4-3 and 4-23 genotypes. In summary, Δ*Kinase* transgenic plants selected for good agronomic performance in the greenhouse, in this study, could not cope with the water stress, and on the contrary, displayed exacerbated drought effects during the water shortage.

## Discussion

The transmembrane RLK, ER, transduces environmental signals through a cascade of MAPKs to control organ growth and size (Meng et al., 2012; Shpak, 2013). In addition, its role in abiotic stress tolerance has been described in *Arabidopsis*, rice, and tomatoes (Masle et al., 2005; van Zanten et al., 2009; Villagarcia et al., 2012; Shen et al., 2015). Suppression of ER signaling in tomato and soybean was linked with drought tolerance, and its overexpression in rice with the heat tolerance (Villagarcia et al., 2012; Shen et al., 2015; Shanmugam et al., 2020). The mechanism of heat tolerance was not clear, but drought tolerance was apparently associated with reduced transpiration surface in the plants (Villagarcia et al., 2012; Shanmugam et al., 2020). However, since ER suppression leads to diminished growth (Torii et al., 2003; Shpak et al., 2004), this study investigated whether drought tolerance through ER suppression could be gained without disturbing desirable agronomic traits in the crop plants such as soybean. Toward this, soybean plants expressing *Arabidopsis ER* gene carrying deletion in the kinase domain (Δ*Kinase*), thus casting dominant-negative effects on ER signaling, were evaluated for desirable agronomic traits in the greenhouse.

Among the plants derived from three different transgenic events expressing Δ*Kinase*, a range of phenotype was observed. These plants generally showed different plant height and leaf sizes and numbers. These characteristics appeared to correlate with plant’s rate of water consumption during the vegetative growth. Stress tolerance in plants, however, must be combined with the favorable traits for agricultural production. A cultivar that is tolerant to stress but not agriculturally superior has no commercial value. Therefore, Δ*Kinase* soybean plants were subjected to the analysis of agronomic performance in the greenhouse. Although the ideal cultivar for each crop may vary regionally, the final objective of plant selection is to increase the productivity potential. Thus, simultaneous selection of a set of traits of agronomic importance serves as an optimum approach. One of the forms of simultaneous selection involving multiple traits is the use of selection index. The SR index is a non-parametric index that is proven satisfactory in the selection of superior soybean lines through recurrent selection (Garcia and Souza Júnior, 1999; Freiria et al., 2019).

The Δ*Kinase* confers a dominant-negative effect by suppressing ER signaling and generates a suppressive effect on organ size and plant architecture (Shpak et al., 2003, 2005). Accordingly, among the 10 plants selected for good agronomic characteristics, six had null or near-null expression for the Δ*Kinase* gene. Evaluating Δ*Kinase*-expressing tomato (*Solanum lycopersicum*) plants, Villagarcia et al. (2012) reported reduction in the size and number of leaves, which resulted in lower dry weight of shoot and roots, when compared with the WT. However, no penalty for the size and number of fruits was observed in the tomato plants. In contrast, *A. thaliana* showed inverse relation between Δ*Kinase* expression and the impact on the phenotype, showing a correlation of Δ*Kinase* expression with shorter stature of plants and penalty for inflorescence, including shorter siliques and reduced fertility (Shpak et al., 2003). Despite this, in the present study, it was possible to identify three lines that were associated with high performance and moderate levels of Δ*Kinase* expression. This indicates, in principle, the possibility of selecting Δ*Kinase* transgenic plants with the agronomic ideotype for genetic improvement of the species. It also highlights the importance of phenotyping in early generations for reducing cost and time in the development of transgenic cultivars. In order to obtain agronomically superior, drought-tolerant plants, progeny plants from the two selected lines (4-3 and 4-23) were analyzed for their response to water deficit.

In soybean genome, four *GmER* and four *GmER-LIKE* genes have been identified (Zhang et al., 2018). The four *GmER* homologs possibly play distinct roles in different tissues or organs of soybean (Du et al., 2017). In the present work, higher expression of Δ*Kinase* was observed in the young leaves than in the young pods. However, although Du et al. (2017) described a higher level of expression of the four *GmERs* upon the exposure of soybean seedlings to dehydration, we did not observe a pattern of difference in Δ*Kinase* expression in the leaves of plants under normal or water-deficit condition. Therefore, Δ*Kinase* does not seem to be regulated by water stress.

Soybean is susceptible to drought, especially during the reproductive stages. Cellular dehydration leading to the loss of turgor is one of the first biophysical effects of dehydration stress (Bartlett et al., 2012). In our study, the selected Δ*Kinase*-expressing plants were found to be more sensitive to drought as compared with the WT, showing dramatic loss of turgor in just 1 day. Transpiration through the open stomata is the main means by which plants lose water to the atmosphere. The replacement of water must be at adequate levels to maintain the hydration of tissues in the plant (Kramer and Boyer, 1995). Leaves are mostly responsible for transpiration, and the leaf area index (LAI), defined as the area of foliage per unit ground surface, plays an important role in the transpiration model and estimation of water loss (Wang et al., 2017). The selected transgenic soybean plants from genotypes 4-3 and 4-23 showed a higher vegetative growth as compared with the WT, which was maintained until the end of the cycle resulting in a higher biomass production under normal irrigation conditions. Since the grain yield and number of nodes in the harvest did not differ between the treatments, the observed shoot biomass in the transgenic plants can be associated with greater size or number of leaves. Thus, transgenic plants of these two lines had a larger leaf area and, consequently, increased transpiration. As a result, they demanded a greater amount of water as compared with the WT. Although Shanmugam et al. (2020) have observed greater tolerance of transgenic Δ*Kinase* plants to water deficit, their plants were not selected through agronomic performance analysis, and it is unclear whether those plants represent the phenotypic ideotype for the genetic improvement of soybean.

Stomatal density as well as stoma opening during CO_2_ absorption plays important roles in controlling water loss by plants during the water-deficit period. In this study, we observed greater stomatal conductance in the well-irrigated transgenic plants but no difference in the water-stressed plants. Similarly, in tomato-expressing Δ*Kinase*, no significant difference was found in the plants subjected to stress. Accordingly, drought tolerance in Δ*Kinase* tomato was assigned to smaller leaf area and lower transpiration surface (Villagarcia et al., 2012). These findings and our study suggest an inverse relationship between shoot biomass and stress tolerance. Furthermore, the suppression of the ER signaling in our soybean plants led to an increase in the number of stomata, which may have contributed to rapid loss of water in the leaves of the transgenic plants. Shpak et al. (2005), studying the roles of ER family protein in epidermal cell patterning, observed increasing stomata density in the single and combination *er* and *erl* mutants of *Arabidopsis*.

In conclusion, the suppression of ER signaling, in this study, led to the loss of favorable phenotypic traits in soybean, including plant biomass and grain yield, and the plants selected through phenotypic performance analysis did not show tolerance to water deficit stress. Therefore, development of drought-tolerant cultivars through the modulation of ER signaling may require alternative approaches such as spatiotemporal suppression/overexpression or the modification of agonist molecules that trigger the downstream signaling.

## Data Availability Statement

The original contributions presented in the study are included in the article/Supplementary Material, further inquiries can be directed to the corresponding author/s.

## Author Contributions

YB and FB conducted the experiments. FB and VS designed the project. All authors participated in data analysis and manuscript writing.

## Conflict of Interest

The authors declare that the research was conducted in the absence of any commercial or financial relationships that could be construed as a potential conflict of interest.

## References

[B1] Babicki S. Arndt D. Marcu A. Liang Y. Grant J. R. Maciejewski A. (2016). Heatmapper: web-enabled heat mapping for all. *Nucleic Acids Res.* 44 147–153. 10.1093/nar/gkw419 27190236 PMC4987948

[B2] Bartlett M. K. Scoffoni C. Sack L. (2012). The determinants of leaf turgor loss point and prediction of drought tolerance of species and biomes: a global meta−analysis. *Ecol. Lett.* 15 393–405. 10.1111/j.1461-0248.2012.01751.x 22435987

[B3] Desclaux D. Huynh T. T. Roumet P. (2000). Identification of soybean plant characteristics that indicate the timing of drought stress. *Crop Sci.* 40 716–722. 10.2135/cropsci2000.403716x

[B4] Du J. Sun X. Sun M. Jiang H. Li Y. Wan C. (2017). Identification and expression analysis of ERECTA homologous genes in Glycine max. *Int. J. Agric. Biol.* 19 1497–1504. 10.17957/ijab/15.0449 29653435

[B5] Fehr W. R. Caviness C. E. (1977). *Stages of Soybean Development. Iowa Coop. Ext. Serv. Special. Rep. 80.* Ames, IA: Iowa State University.

[B6] Freiria G. H. Perini L. J. Zeffa D. M. Novais P. S. Lima W. F. Gonçalves L. S. A. (2019). Comparison of non-parametric indexes to select soybean genotypes obtained by recurrent selection. *Semina Ciênc. Agrár.* 40 1761–1774. 10.5433/1679-0359.2019v40n5p1761

[B7] Garcia A. A. F. Souza Júnior C. L. (1999). Comparação de índices de seleção não paramétricos para a seleção de cultivares. *Bragantia* 58 253–267.

[B8] Kaur N. Gupta A. K. (2005). Signal transduction pathways under abiotic stresses in plants. *Curr. Sci.* 88 1771–1780.

[B9] Kramer P. J. Boyer J. S. (1995). *Water Relations of Plants and Soils.* Cambridge, MA: Academic press.

[B10] Kunert K. J. Vorster B. J. Fenta B. A. Kibido T. Dionisio G. Foyer C. H. (2016). Drought stress responses in soybean roots and nodules. *Front. Plant Sci.* 7:1015. 10.3389/fpls.2016.01015 27462339 PMC4941547

[B11] Leng G. Hall J. (2019). Crop yield sensitivity of global major agricultural countries to droughts and the projected changes in the future. *Sci. Total Environ.* 654 811–821. 10.1016/j.scitotenv.2018.10.434 30448671 PMC6341212

[B12] Lesk C. Rowhani P. Ramankutty N. (2016). Influence of extreme weather disasters on global crop production. *Nature* 529 84–87. 10.1038/nature16467 26738594

[B13] Livak K. J. Schmittgen T. D. (2001). Analysis of relative gene expression data using real-time quantitative PCR and the 2-DDCT method. *Methods* 25 402–408. 10.1006/meth.2001.1262 11846609

[B14] Masle J. Gilmore S. R. Farquha G. D. (2005). The ERECTA gene regulates plant transpiration efficiency in *Arabidopsis*. *Nature* 436 866–870. 10.1038/nature03835 16007076

[B15] Meng X. Wang H. He Y. Liu Y. Walker J. C. Torii K. U. (2012). A MAPK cascade downstream of ERECTA receptor-like protein kinase regulates *Arabidopsis* inflorescence architecture by promoting localized cell proliferation. *Plant Cell* 24 4948–4960. 10.1105/tpc.112.104695 23263767 PMC3556968

[B16] Mulamba N. N. Mock J. J. (1978). Improvement of yield potential of the method Eto Blanco maize (*Zea mays* L.) population by breeding for plant traits. *Egypt. J. Genet. Cytol.* 7 40–51.

[B17] Nguyen H. C. Lin K. H. Hsiung T. C. Huang M. Y. Yang C. M. Weng J. H. (2018). Biochemical and physiological characteristics of photosynthesis in plants of two *Calathea* species. *Int. J. Mol. Sci* 19:704. 10.3390/ijms19030704 29494547 PMC5877565

[B18] Oya T. Nepomuceno A. L. Neumaier N. Farias J. R. B. Tobita S. Ito O. (2004). Drought tolerance characteristics of Brazilian soybean cultivars. *Plant Prod. Sci.* 7 129–137. 10.1626/pps.7.129 33707419

[B19] R Core Team (2017). *R: A Language and Environment for Statistical Computing.* R Foundation for Statistical Computing, Available on;line at: http://www.R-project.org (accessed July 20, 2020).

[B20] RStudio Team (2016). *RStudio: Integrated Development for R.* Boston, MA: RStudio, Inc,

[B21] Shanmugam S. Zhao S. Nandy S. Srivastava V. Khodakovskaya M. (2020). Modification of soybean growth and abiotic stress tolerance by expression of truncated ERECTA protein from *Arabidopsis thaliana*. *PLoS One* 15:e0233383. 10.1371/journal.pone.0233383 32428035 PMC7236981

[B22] Shen H. Zhong X. Zhao F. Wang Y. Yan B. Li Q. (2015). Overexpression ofreceptor-like kinase ERECTA improves thermotolerance in rice and *Tomato*. *Nat. Biotechnol.* 33 996–1003. 10.1038/nbt.3321 26280413

[B23] Shpak E. D. (2013). Diverse roles of ERECTA family genes in plant development. *J. Intr. Plant Biol.* 55 1238–1250. 10.1111/jipb.12108 24016315

[B24] Shpak E. D. Berthiaume C. D. Hill E. J. Torii K. U. (2004). Synergistic interaction of three ERECTA-family receptor-like kinases controls *Arabidopsis* organ growth and flower development by promoting cell proliferation. *Development* 131 1491–1501. 10.1242/dev.01028 14985254

[B25] Shpak E. D. Lakeman M. B. Torii K. U. (2003). Dominant-negative receptor uncovers redundancy in the *Arabidopsis* ERECTA Leucine-rich repeat receptor-like kinase signaling pathway that regulates organ shape. *Plant Cell* 15 1095–1110. 10.1105/tpc.010413 12724536 PMC153719

[B26] Shpak E. D. McAbee J. M. Pillitteri L. J. Torii K. U. (2005). Stomatal patterning and differentiation by synergistic interactions of receptor kinases. *Science* 2005 290–293. 10.1126/science.1109710 16002616

[B27] Sinha A. K. Jaggi M. Raghuram B. Tuteja N. (2011). Mitogen-activated protein kinase signaling in plants under abiotic stress. *Plant Signal. Behav.* 6, 196–203. 10.4161/psb.6.2.1470 21512321 PMC3121978

[B28] Torii K. U. Hanson L. A. Josefsson C. A. Shpak E. D. (2003). “Regulation of inflorescence architecture and organ shape by the ERECTA gene in *Arabidopsis*,” in *Morphogenesis and Patterning in Biological Systems*, ed. Sekimura T. (Tokyo: Springer−Verlag), 153–164. 10.1007/978-4-431-65958-7_13

[B29] Torii K. U. Mitsukawa N. Oosumi T. Matsuura Y. Yokoyama R. Whittier R. F. (1996). The *Arabidopsis* ERECTA gene encodes a putative receptor protein kinase with extracellular leucine-rich repeats. *Plant Cell* 8 735–746. 10.1105/tpc.8.4.735 8624444 PMC161133

[B30] van Zanten M. Snoek L. B. Proveniers M. C. Peeters A. J. (2009). The many functions of ERECTA. *Trend. Plant Sci.* 14 214–218. 10.1016/j.tplants.2009.01.010 19303350

[B31] Villagarcia H. Morin A. C. Shpak E. D. Khodakovskaya M. V. (2012). Modification of tomato growth by expression of truncated ERECTA protein from *Arabidopsis thaliana*. *J. Exp. Bot.* 63 6493–6504. 10.1093/jxb/ers305 23096000

[B32] Wang H. Sánchez-Molina J. A. Li M. Berenguel M. Yang X. T. Bienvenido J. F. (2017). Leaf area index estimation for a greenhouse transpiration model using external climate conditions based on genetics algorithms, back-propagation neural networks and nonlinear autoregressive exogenous models. *Agric. Water. Manag.* 183 107–115. 10.1016/j.agwat.2016.11.021

[B33] Xu C. Xia C. Xia Z. Zhou X. Huang J. Huang Z. (2018). Physiological and transcriptomic responses of reproductive stage soybean to drought stress. *Plant Cell Rep.* 37 1611–1624. 10.1007/s00299-018-2332-3 30099610

[B34] Zhang Y. Li S. Xue S. Yang S. Huang J. Wang L. (2018). Phylogenetic and CRISPR/Cas9 studies in deciphering the evolutionary trajectory and phenotypic impacts of rice ERECTA genes. *Front. Plant Sci.* 9:473. 10.3389/fpls.2018.00473 29692796 PMC5902711

