# Supplementary material for: Suppression of ERECTA Signaling Impacts Agronomic Performance of Soybean (Glycine max (L) Merril) in the Greenhouse
Source: Front Plant Sci. 2021 May 11;12:667825. doi: 10.3389/fpls.2021.667825 (PMC8148577; doi:10.3389/fpls.2021.667825)
Supplement: Supplementary file 1 [file Table_1.DOCX]

**Supplementary Table 1.** Number of plants and lines evaluated during three generations of transgenic soybean plants expressing *ΔKinase* gene.

| **T_1_ lines** | **T_2_ lines** | **T_2_ ID** | **Number T_3_ lines** |
| --- | --- | --- | --- |
| Wild-type (WT) | ST 301 -WT | **1** | 3 |
| 1 | ST 301 - 1-1 | **2** | 1 |
|  | ST 301 - 1-2 | **3** | 6 |
|  | ST 301 - 1-4 | **4** | 16 |
| 2 | ST 301 - 2-1 | **5** | 10 |
|  | ST 301 - 2-2 | **6** | 2 |
|  | ST 301 - 2-4 | **7** | 8 |
|  | ST 301 - 2-6 | **8** | 8 |
|  | ST 301 - 2-7 | **9** | 5 |
| 3 | ST 301 - 3-1 | **10** | 12 |
|  | ST 301 - 3-4 | **11** | 11 |
| Empty vector (EV) | EV | **12** | 11 |
