# Supplementary material for: Suppression of ERECTA Signaling Impacts Agronomic Performance of Soybean (Glycine max (L) Merril) in the Greenhouse
Source: Front Plant Sci. 2021 May 11;12:667825. doi: 10.3389/fpls.2021.667825 (PMC8148577; doi:10.3389/fpls.2021.667825)
Supplement: Supplementary file 2 [file Table_2.DOCX]

**Supplementary Table 2.** T_3_ progenies ranked by Sum of Rank (SR) index

| **Rank** | **Progeny** | **SR** | **Rank** | **Progeny** | **SR** | **Rank** | **Progeny** | **SR** |
| --- | --- | --- | --- | --- | --- | --- | --- | --- |
| **1** | 12-20 | 97 | **33** | 4-2 | 245 | **65** | 10-11 | 352 |
| **2** | 4-23 | 98 | **34** | 7-23 | 245 | **66** | 9-6 | 356 |
| **3** | 12-22 | 109 | **35** | 12-7 | 252 | **67** | 10-9 | 357 |
| **4** | 4-3 | 115 | **36** | 8-9 | 256 | **68** | 10-16 | 359 |
| **5** | 1-18 | 117 | **37** | 10-18 | 257 | **69** | 9-28 | 365 |
| **6** | 4-12 | 130 | **38** | 11-2 | 259 | **70** | 6-17 | 367 |
| **7** | 12-4 | 130 | **39** | 7-19 | 259 | **71** | 5-23 | 369 |
| **8** | 12-10 | 131 | **40** | 11-10 | 271 | **72** | 10-6 | 382 |
| **9** | 4-18 | 149 | **41** | 5-17 | 277 | **73** | 5-12 | 387 |
| **10** | 12-12 | 152 | **42** | 3-27 | 280 | **74** | 7-13 | 395 |
| **11** | 11-13 | 152 | **43** | 4-5 | 283 | **75** | 8-30 | 403 |
| **12** | 4-6 | 161 | **44** | 7-24 | 283 | **76** | 10-1 | 408 |
| **13** | 12-1 | 172 | **45** | 4-8 | 286 | **77** | 7-9 | 419 |
| **14** | 3-28 | 174 | **46** | 10-7 | 288 | **78** | 11-22 | 420 |
| **15** | 12-23 | 178 | **47** | 1-19 | 295 | **79** | 8-25 | 424 |
| **16** | 12-28 | 178 | **48** | 5-16 | 295 | **80** | 8-17 | 429 |
| **17** | 9-21 | 178 | **49** | 1-27 | 296 | **81** | 7-16 | 432 |
| **18** | 4-4 | 184 | **50** | 8-2 | 297 | **82** | 7-4 | 434 |
| **19** | 11-7 | 187 | **51** | 11-3 | 298 | **83** | 5-13 | 446 |
| **20** | 12-24 | 194 | **52** | 2-30 | 300 | **84** | 7-29 | 454 |
| **21** | 3-29 | 196 | **53** | 10-4 | 311 | **85** | 9-18 | 458 |
| **22** | 11-12 | 202 | **54** | 10-5 | 312 | **86** | 8-8 | 469 |
| **23** | 4-1 | 206 | **55** | 8-18 | 315 | **87** | 5-11 | 476 |
| **24** | 11-15 | 208 | **56** | 11-11 | 318 | **88** | 6-14 | 478 |
| **25** | 11-5 | 223 | **57** | 3-1 | 318 | **89** | 5-15 | 481 |
| **26** | 3-26 | 224 | **58** | 11-20 | 319 | **90** | 7-26 | 488 |
| **27** | 4-25 | 224 | **59** | 4-17 | 319 | **91** | 8-27 | 495 |
| **28** | 3-2 | 226 | **60** | 4-21 | 335 | **92** | 9-16 | 505 |
| **29** | 5-25 | 226 | **61** | 10-19 | 337 | **93** | 10-29 | 505 |
| **30** | 4-19 | 231 | **62** | 4-20 | 341 | **94** | 5-27 | 514 |
| **31** | 12-8 | 242 | **63** | 4-10 | 349 |  |  |  |
| **32** | 10-25 | 245 | **64** | 5-26 | 351 |  |  |  |
