# Supplementary figures and images for: Suppression of ERECTA Signaling Impacts Agronomic Performance of Soybean (Glycine max (L) Merril) in the Greenhouse
Source: Front Plant Sci. 2021 May 11;12:667825. doi: 10.3389/fpls.2021.667825 (PMC8148577; doi:10.3389/fpls.2021.667825)

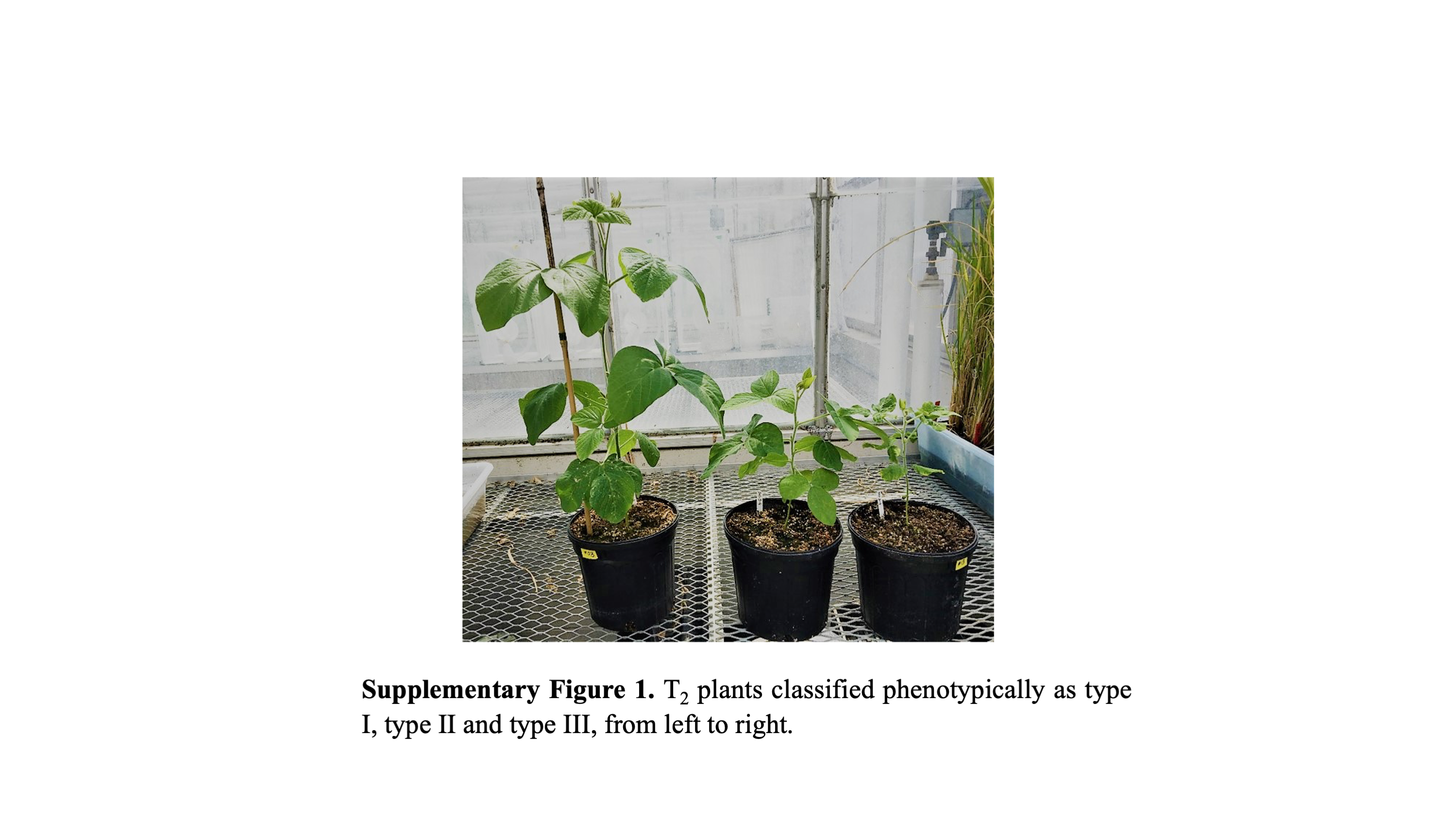

Supplement: Supplementary file 3 [file Image_1.TIFF]

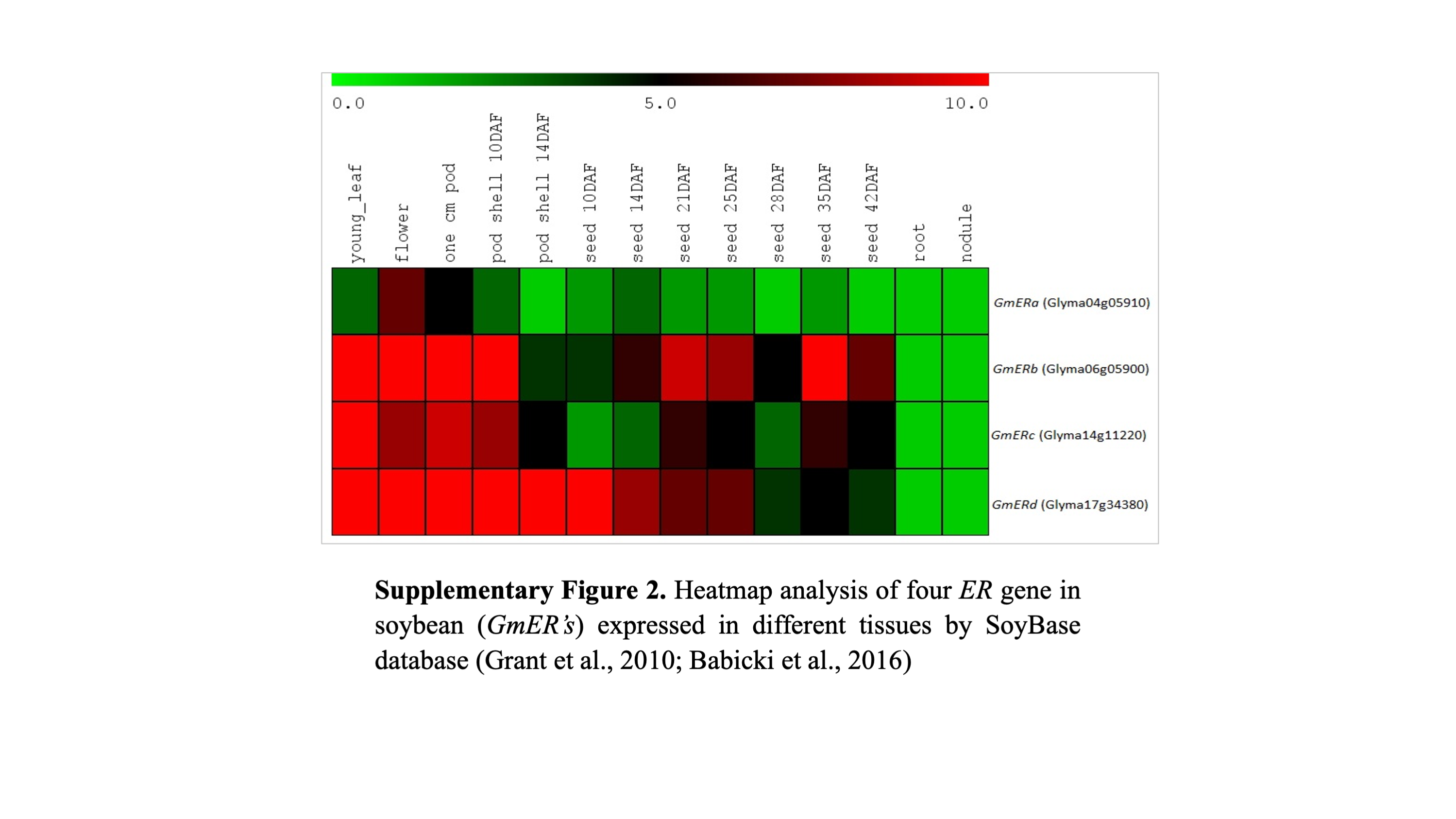

Supplement: Supplementary file 4 [file Image_2.TIFF]

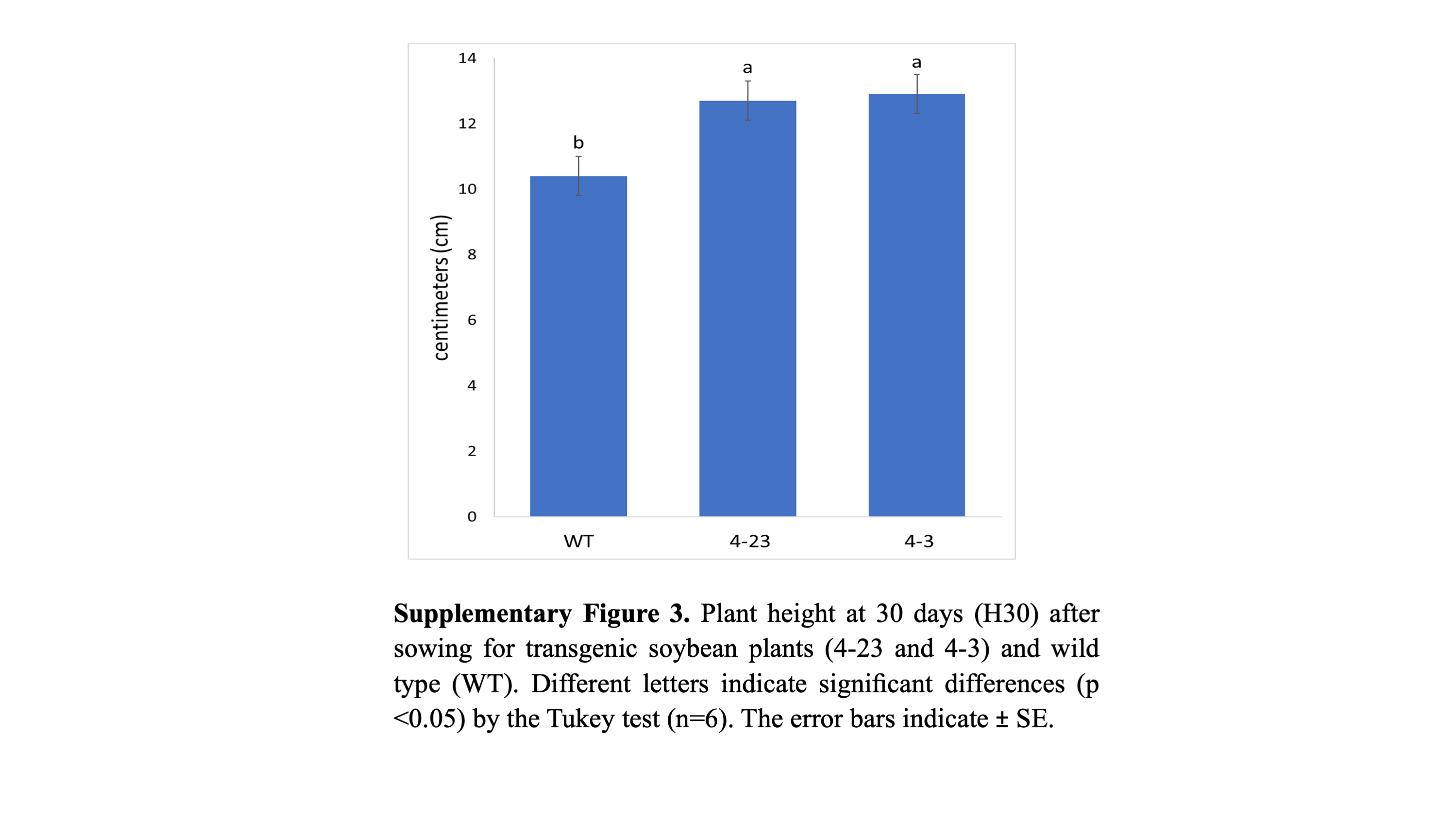

Supplement: Supplementary file 5 [file Image_3.TIFF]

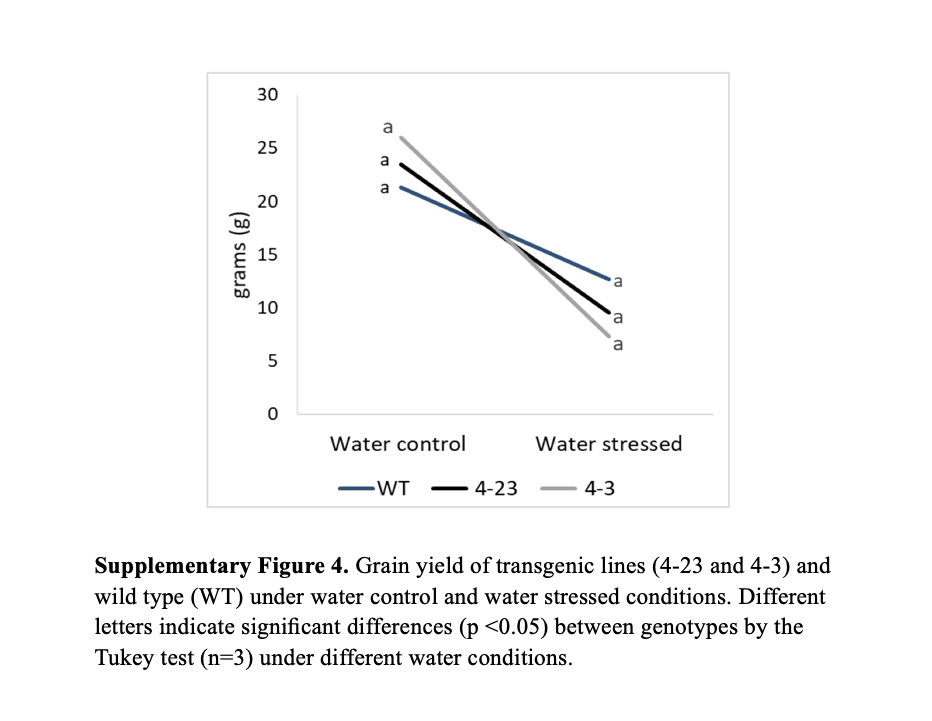

Supplement: Supplementary file 6 [file Image_4.TIFF]

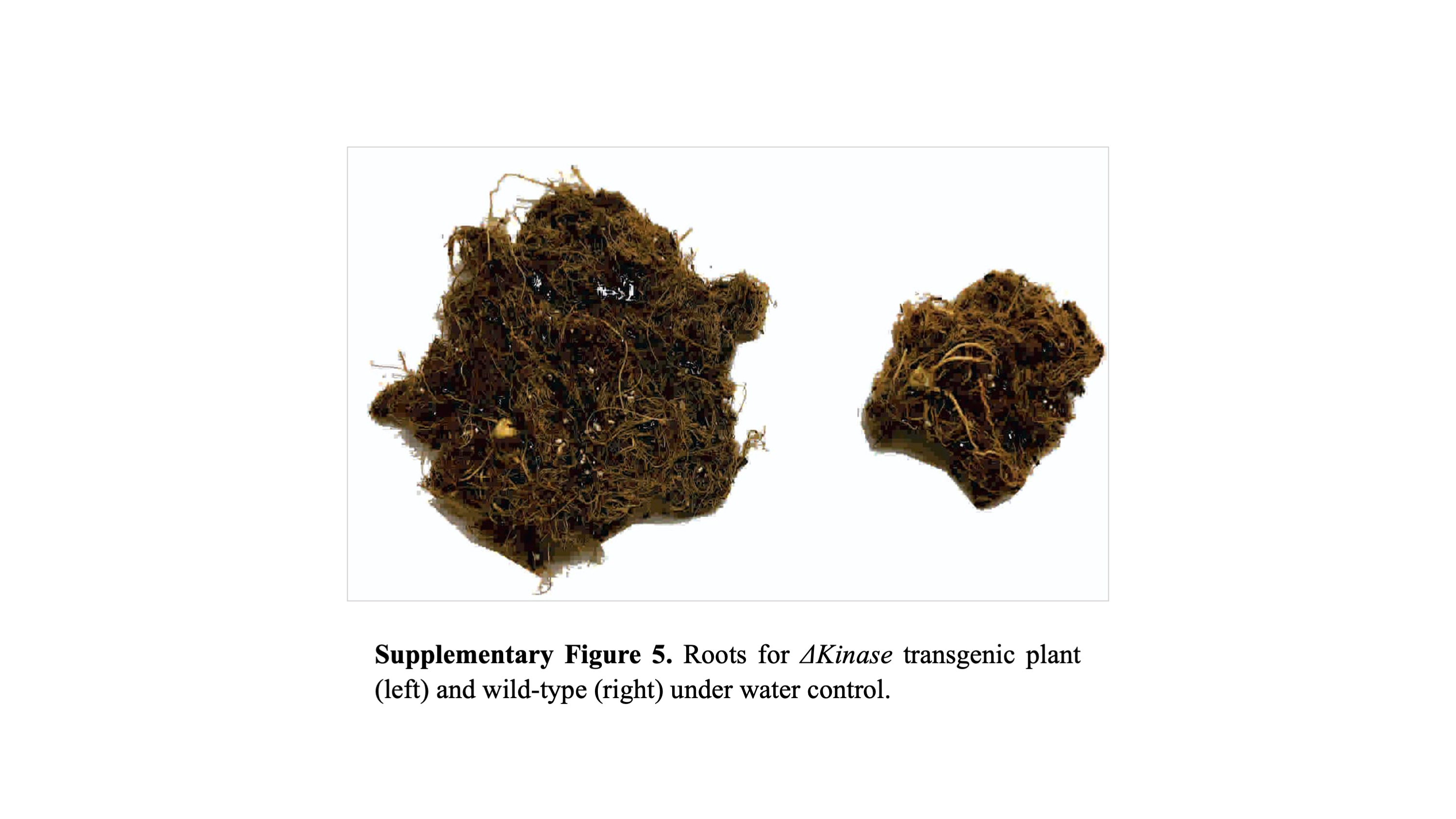

Supplement: Supplementary file 7 [file Image_5.TIFF]
